# Delta-9-tetrahydrocannabinol, neural oscillations above 20 Hz and induced acute psychosis

**DOI:** 10.1007/s00213-014-3684-1

**Published:** 2014-07-20

**Authors:** Judith F. Nottage, James Stone, Robin M. Murray, Alex Sumich, Elvira Bramon-Bosch, Dominic ffytche, Paul D. Morrison

**Affiliations:** 1 Institute of Psychiatry, King’s College London, P089 DeCrespigny Park, Denmark Hill, London, SE5 8AF UK; 2 Nottingham Trent University, Nottingham, NG1 4BU UK; 3 University College London, London, UK

**Keywords:** THC, EEG, Beta, Gamma, Psychosis, Cannabis

## Abstract

**Rationale:**

An acute challenge with delta-9-tetrahydrocannabinol (THC) can induce psychotic symptoms including delusions. High electroencephalography (EEG) frequencies, above 20 Hz, have previously been implicated in psychosis and schizophrenia.

**Objectives:**

The objective of this study is to determine the effect of intravenous THC compared to placebo on high-frequency EEG.

**Methods:**

A double-blind cross-over study design was used. In the resting state, the high-beta to low-gamma magnitude (21–45 Hz) was investigated (*n* = 13 pairs + 4 THC only). Also, the event-related synchronisation (ERS) of motor-associated high gamma was studied using a self-paced button press task (*n* = 15).

**Results:**

In the resting state, there was a significant condition × frequency interaction (*p* = 0.00017), consisting of a shift towards higher frequencies under THC conditions (reduced high beta [21–27 Hz] and increased low gamma [27–45 Hz]). There was also a condition × frequency × location interaction (*p* = 0.006), such that the reduction in 21–27-Hz magnitude tended to be more prominent in anterior regions, whilst posterior areas tended to show greater 27–45-Hz increases. This effect was correlated with positive symptoms, as assessed on the Positive and Negative Syndrome Scale (PANSS) (*r* = 0.429, *p* = 0.042). In the motor task, there was a main effect of THC to increase 65–130-Hz ERS (*p* = 0.035) over contra-lateral sensorimotor areas, which was driven by increased magnitude in the higher, 85–130-Hz band (*p* = 0.02) and not the 65–85-Hz band.

**Conclusions:**

The THC-induced shift to faster gamma oscillations may represent an over-activation of the cortex, possibly related to saliency misattribution in the delusional state.

**Electronic supplementary material:**

The online version of this article (doi:10.1007/s00213-014-3684-1) contains supplementary material, which is available to authorized users.

## Introduction

There is evidence that the chronic use of ∆9-tetrahydrocannabinol (THC) is a risk factor for schizophrenia (Andreasson et al. 1987; Arseneault et al. 2002; Henquet et al. 2005; Moore et al. 2007). Moreover, acute administration of ∆9-THC has been shown to produce transient psychotic symptoms in healthy subjects, particularly delusions, (D’Souza et al. 2004; Morrison et al. 2009), thus modelling some of the symptoms of schizophrenia (Luzi et al. 2008; D’Souza et al. 2009; Koethe et al. 2009).

Neural oscillations, which are repetitive increases and decreases in neuronal or synaptic activity, are known to be important in brain function and to generate waves in the electrical fields which can be detected at the scalp using electroencephalography (EEG). EEG oscillations can be measured in three main ways: Firstly, phase-locking of oscillations to an event, such as a stimulus presentation or response, can be measured using event-related potentials, inter-trial coherence, evoked magnitude or steady-state responses to a rapid stimulus train. Secondly, resting-state magnitude of the waves can be recorded without reference to any events. Thirdly, changes in magnitude, due to an event, can be quantified ignoring phase locking. In this case, an increase in magnitude is termed an event-related synchronisation (ERS) and a decrease in magnitude is an event-related desynchronisation (ERD). Cannabinoids have been reported to alter the EEG signal. Firstly, THC tends to reduce phase-related measures; there is a dose-dependent reduction of the P300 with acute THC (D’Souza et al. 2012) and reduced inter-trial coherence. (Stone et al. 2012). Furthermore, in chronic cannabis users, Auditory Steady-State Responses (ASSRs) to 40-Hz click trains have been found to be reduced (Skosnik et al. 2012), demonstrating an impairment in the capacity of neural circuits to support gamma band oscillations. These changes are similar to reductions in event-related potentials and phase locking observed in schizophrenia (Kwon et al. 1999; Bramon et al. 2005; Spencer et al. 2008; Hall et al. 2011), although there have been contradictory results (Spencer and Ghorashi 2014). THC also causes changes in neural oscillations independent of phase locking to an event. In humans, acute THC has been shown to reduce the magnitude and coherence of lower frequencies of the human EEG spectra (Ilan et al. 2005; Zuurman et al. 2008; Bocker et al. 2010; Morrison et al. 2011; Stone et al. 2012). Moreover, animal studies have demonstrated that cannabinoid-1 (CB1) agonists, such as ∆9-THC, significantly affect neuronal oscillations in the gamma band (>30 Hz) (Robbe et al. 2006; Hajos et al. 2008). In psychotic disorders, including schizophrenia, both increases and decreases in EEG magnitude at rest have been reported in psychotic disorders including schizophrenia (Giannitrapani and Kayton 1974; Itil 1977; Venables et al. 2009; Ranlund et al. 2014). Changes have also been observed in task-related gamma band activity, such as decreased ERS in an auditory task occurring at frontal and left-sided locations but increased ERS in posterior and right locations (Haig et al. 2000). Synchrony between different brain areas is also altered above 20 Hz, being reduced at 20–30 Hz in schizophrenia in a Gestalt task (Uhlhaas et al. 2006) but increased at rest in temporal areas in the 35–45-Hz low-gamma (LG) band (Flynn et al. 2008). More recently task-related deficits at high-gamma (HG) frequencies (>60 Hz) have been observed using MEG (Uhlhaas 2011; Uhlhaas et al. 2011). Since aberrant salience attribution is thought to underlie delusions (Kapur 2003) and a higher peak frequency of gamma may reflect the saliency of a stimulus (van Pelt and Fries 2013), the frequency distribution of high-frequency oscillations may be important in psychosis, but this has not yet been investigated.

Cannabinoids could alter cortical oscillations either via activation of cortical receptors or indirectly via sub-cortical structures, such as the basal ganglia. THC acts mainly on CB1 receptors, which are particularly dense in the components of cortico-striatal circuits (Herkenham et al. 1990). The main areas involved in the limbic, or reward, cortico-striatal circuit are the nucleus accumbens, prefrontal cortical areas and temporal areas including the hippocampus (Alexander et al. 1986; Haber and Knutson 2010), whereas the putamen and motor cortical areas are key nodes in the motor striatal circuit, with the cerebellum being a key target area (Alexander et al. 1986). There is evidence that functional dysconnectivity in cortico-striatal circuitry is an important risk phenotype for psychosis (Dandash et al. 2014; Fornito et al. 2013), whilst antipsychotics are thought to act by antagonism of the dopamine D2 receptors in these circuits. Stimulation of dopamine receptors using l-DOPA can alleviate the motor symptoms of Parkinson’s but also generate psychotic symptoms as a side effect, whilst antipsychotics can give rise to parkinsonian symptoms as side effects. Understanding the effects of THC on these circuits may be key to understanding the mechanism by which THC generates psychosis. Out of the sub-components of these circuits, only some areas of neocortex are close enough to the surface of the brain for the low-amplitude high-frequency waves to be clearly detected on the scalp using EEG. For example, in a self-paced motor task, ERS occurs at high-gamma power which can be detected in the EEG, mainly at 65–85 Hz, but extending up to 130 Hz (Crone et al. 1998; Cheyne et al. 2008; Nottage et al. 2013). Limbic and associative cortico-striatal circuits, which are of relevance to psychosis, will be active during internal thought processes in the resting state, but the exact timing of such activity is unknown, precluding the use of the more sensitive event-related EEG measures. However, since dopamine induces similar effects in all striatal circuits, THC might also act in a similar way. Thus, changes in the motor gamma signal, which can be readily measured, could shed light on the general effects of THC on high-frequency oscillations in cortico-striatal circuits. Whereas the motor high-gamma activity is localised to the contra-lateral motor and somatosensory cortex, resting-state activity is more widely distributed, whilst high-frequency topographic effects of THC are unknown. It is of interest that frontal cortical regions have higher CB1 expression than posterior regions (Glass et al. 1997), the neural circuits through the striatum and thalamus to the cortex project mainly to frontal areas (Herrero et al. 2002), and altered CB1 signalling in prefrontal areas may be important in schizophrenia (Dalton et al. 2011). Also, since changes in gamma ERS in opposite directions in anterior-posterior and posterior regions are reported in schizophrenia (Haig et al. 2000), we asked whether the effect of THC on high-frequency oscillations in the frontal cortex would be different to its effect on posterior cortical areas.

There has been considerable recent concern about the contamination of frequencies above 20 Hz by muscle activity from the scalp, neck and eyes (Goncharova et al. 2003; Whitham et al. 2007; Yuval-Greenberg et al. 2008). In response to this concern, novel artefact reduction techniques have been developed, both to deal with extra-ocular muscle activity (Keren et al. 2010; Nottage 2010; Hassler et al. 2011) and contamination from scalp and neck muscles (Nottage et al. 2013). It has been demonstrated that these can increase the signal to noise ratio of the motor gamma ERS. Therefore, whilst the effect of THC on the resting human EEG spectra up and including 20 Hz has been well established, the effect of acute THC on higher frequencies needs to be clarified using improved artefact correction. A standard frequency band for high beta is 20–30 Hz, whereas the frequencies used for low gamma have varied, usually starting at 30–35 Hz with an upper bound between 40 and 60 Hz. However, increased EEG magnitude confined to a narrow frequency band around 30 Hz has been reported at rest in acute schizophrenia (Giannitrapani and Kayton 1974) and more recently as a rebound ERS after a visual task (Spencer and Ghorashi 2014). Hence, a 30-Hz division between high beta and low gamma is inappropriate here, since it would obscure any such peak. Also, the amplitude of EMG continues to increase with frequency up to at least 60 Hz (Goncharova et al. 2003), whereas the peak of low gamma is thought to be around 40 Hz, so using an upper bound of 45 Hz for the low-gamma analysis would yield a greater signal to noise ratio than using 60 Hz.

We also hypothesised that THC-induced effects would correlate with psychopathology as measured using the Positive and Negative Syndrome Scale (PANSS) (Kay et al. 1987). Since there is evidence that THC acts by exacerbating an underlying predisposition to psychosis (McGuire et al. 1995; Arendt et al. 2008; Barkus and Lewis 2008), it is the final magnitude of the EEG, as opposed to the change induced by THC, that is likely to be most strongly associated with positive symptoms. However, the raw magnitude of resting EEG can vary considerably between subjects, due in part to physical features such as skull thickness. Where changes in frequency or topography are present, the use of ratios of the EEG magnitudes at different frequencies or electrode locations can help reduce this inter-subject variance.

In summary, this study investigates the effect of intravenous THC on resting-state 21–45-Hz magnitude and on the contra-lateral high-gamma 65–130-Hz response to self-paced motor responses. Changes in the frequency distribution of the EEG were considered, as well as changes in the fronto-posterior distribution in the resting state and associations with PANSS positive symptoms.

## Materials and methods

### Subjects

Ethical approval was given by the South London and Maudsley Ethics Committee, and the research was therefore performed in accordance with the ethical standards laid down in the 1964 Declaration of Helsinki. All participants gave written informed consent. A randomised, double-blind, within-participant, experimental design was employed. Intravenous (IV) THC (1.25 mg) or placebo was administered, and resting-state EEG was recorded approximately 10 min post-injection, followed by a self-paced motor task at 20 min post-injection. For 15 subjects, EEG was recorded during a self-paced motor task for THC and placebo sessions. Resting-state EEG was recorded for both THC and placebo sessions for 14 participants and for just the THC session for a further four participants. In one subject, excessive muscle artefact contamination meant that data quality was insufficient to allow processing of high-frequency EEG data, leaving 13 subjects with both THC and placebo resting-state data. Mean age of all participants was 26 ± 6 years, and 59 % were female. Before the experimental sessions, all urine drug screens were negative. Previous use of cannabis ranged from 2 to ~1,000 occasions (median = 40). All participants had used cannabis with previous use of cannabis ranging from 2 to ~4,000 occasions (median = 100). With regard to other drugs, 76 % had previously taken stimulants (cocaine/amphetamines), 41 % had taken psychedelics (psilocybin/LSD), and there was a single case of gamma-hydroxybutyric acid use. All participants had consumed alcohol, and 53 % were smoking tobacco at the time of testing.

### EEG recording

EEG was recorded using a Neuroscan 64 channel Synamps amplifier using the 10–20 system with a linked mastoid reference. Additional electrodes were positioned at the outer corners of the left and right eyes and the power-line noise was also recorded. The sampling rate was 2,000 Hz, resolution was 0.1 μV, and filter settings were 0.05 (high pass) to 200 Hz (low pass).

### Tasks

Resting-state EEG was recorded for at least 2 min, whilst participants were sitting in a chair with arm supports. Every 30 s, a verbal instruction was given to the participant to open or close their eyes. Only the eyes-closed data was included in the analysis, to avoid contamination with artefacts from the eyes. For the self-paced motor task, the participants were instructed to press a button with their index finger, on a game pad located next to their right hand, approximately once per second. After 70 button presses the words “task finished” appeared on the computer screen.

### EEG analysis

Frequencies above 20 Hz were analysed using methods described in detail elsewhere (Nottage 2010; Nottage et al. 2013). In brief, power-line noise was reduced by noise cancellation, and the micro-saccade artefacts were removed using regression in the motor task. The scalp, face and neck EMG was reduced by mathematical modelling and subtraction of individual muscle spikes, for all epochs in which the 60–140-Hz amplitude exceeded 0.03 μV/Hz at any time point. The thresholds for the minimum amplitude of EMG spike were set at 7 μV for <85 Hz and 4 μV for >85 Hz. After artefact correction, sliding windows of 256 ms, 5 ms apart, were cut for high motor gamma (>65 Hz), giving frequency bins 3.91 Hz wide, and 512 ms, 50 ms apart, for high beta/low gamma (21–45 Hz), giving frequency bins 1.95 Hz wide. A frequency-based rejection procedure was used as described previously (Nottage et al. 2013). After linear detrending and applying Hanning windows, FFTs were applied. The initial preprocessing stages were carried out in Neuroscan 4.3, after which the analysis was carried out in MATLAB, including use of the Signal Processing Toolbox.

### Electrode derivations and frequency bands: resting state

**Fig. 1:**
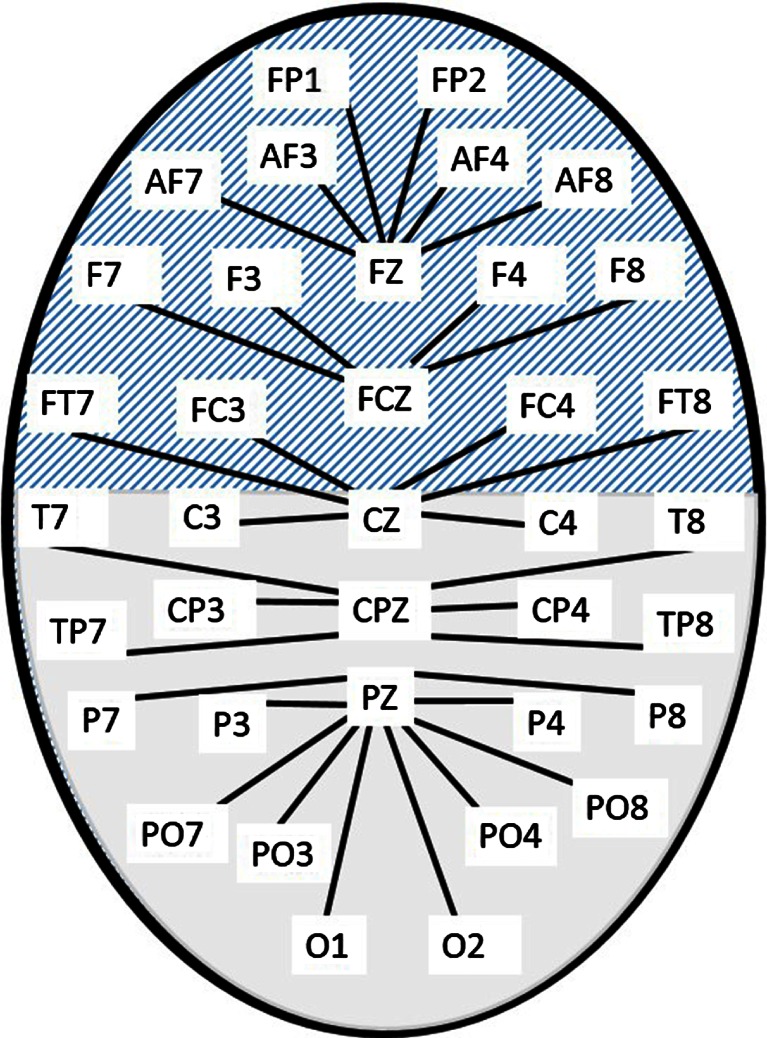
Bipolar montage used for resting-state analysis. The *hatched area* denotes electrodes in the anterior group, and the *solid fill* those in the posterior group. Each *line* corresponds to a bipolar electrode derivation, such as F7-FCZ or O2-PZ

The 21–45-Hz frequency band was divided into three sub-bands, each with a band width of one quarter of its central frequency, giving high beta (21–27 Hz), beta/gamma (27–35 Hz) and low gamma (35–45 Hz). This allowed the analysis to be sensitive to a narrow spectral peak at 30 Hz, as well as other changes in frequency distribution. In order to apply the EMG reduction, there should only be one source of muscle spikes. Therefore, midline electrodes (FZ, FCZ, CZ, PZ and CPZ), which are relatively free from EMG contamination (Fitzgibbon et al. 2013), were used as reference electrodes, to generate 32 bipolar channels, as shown in Fig. 1. These were divided into two groups, anterior and posterior, as shown by the hatched and solid fill areas in Fig. 1, and amplitude values for each group were averaged before further analysis.

### Electrode derivations and frequency bands: motor task

A left central bipolar channel (C3-CZ) was used for the 65–130-Hz band, which was sub-divided into high gamma (65–85 Hz) and very high gamma (85–130 Hz) so that changes in frequency distribution could be detected. The event-related synchronisation (ERS) in the 250 ms following the button press was calculated using the period −550 to −75 ms as the baseline. A button press was excluded if it followed the previous one by less than 875 ms.

### Statistical tests

Statistical analysis was carried out using SPSS statistical analysis software. For the motor gamma ERS, a paired two-tailed *t* test was carried out on the whole 65–130 Hz band. In the case of a significant effect, the frequency distribution of this effect was explored using further *t* tests with the two sub-bands (65–85 Hz and 85–130 Hz), using Bonferroni correction for multiple comparisons. In the resting-state data, a repeated measures ANOVA was carried out with three factors: condition (drug and placebo), location (anterior and posterior) and frequency (20–27, 27–35 and 35–45 Hz). If sphericity assumptions were violated, Huynh-Feldt corrections were applied. Where significant main EEG amplitude effects were observed, Kendall’s tau correlation coefficients were calculated between PANSS positive scores and the amplitudes and tested for significance using two-tailed tests. If interaction effects were uncovered in the ANOVA, ratios of the relevant EEG amplitudes were calculated, in order to minimise subject to subject variance, and used to test for symptom associations. Bonferroni correction for multiple comparisons was used where necessary. To determine whether there was a specific association between the electrophysiological effects and delusions, a sub-group of the PANSS scores, consisting of those primarily associated with delusions (P1 + P5 + P6: delusions, grandiosity and persecution/suspiciousness), was also tested.

In the resting EEG, the effect of THC on frequencies below 20 Hz was also analysed and is presented in Supplement 1.

## Results

### Resting state

**Fig. 2:**
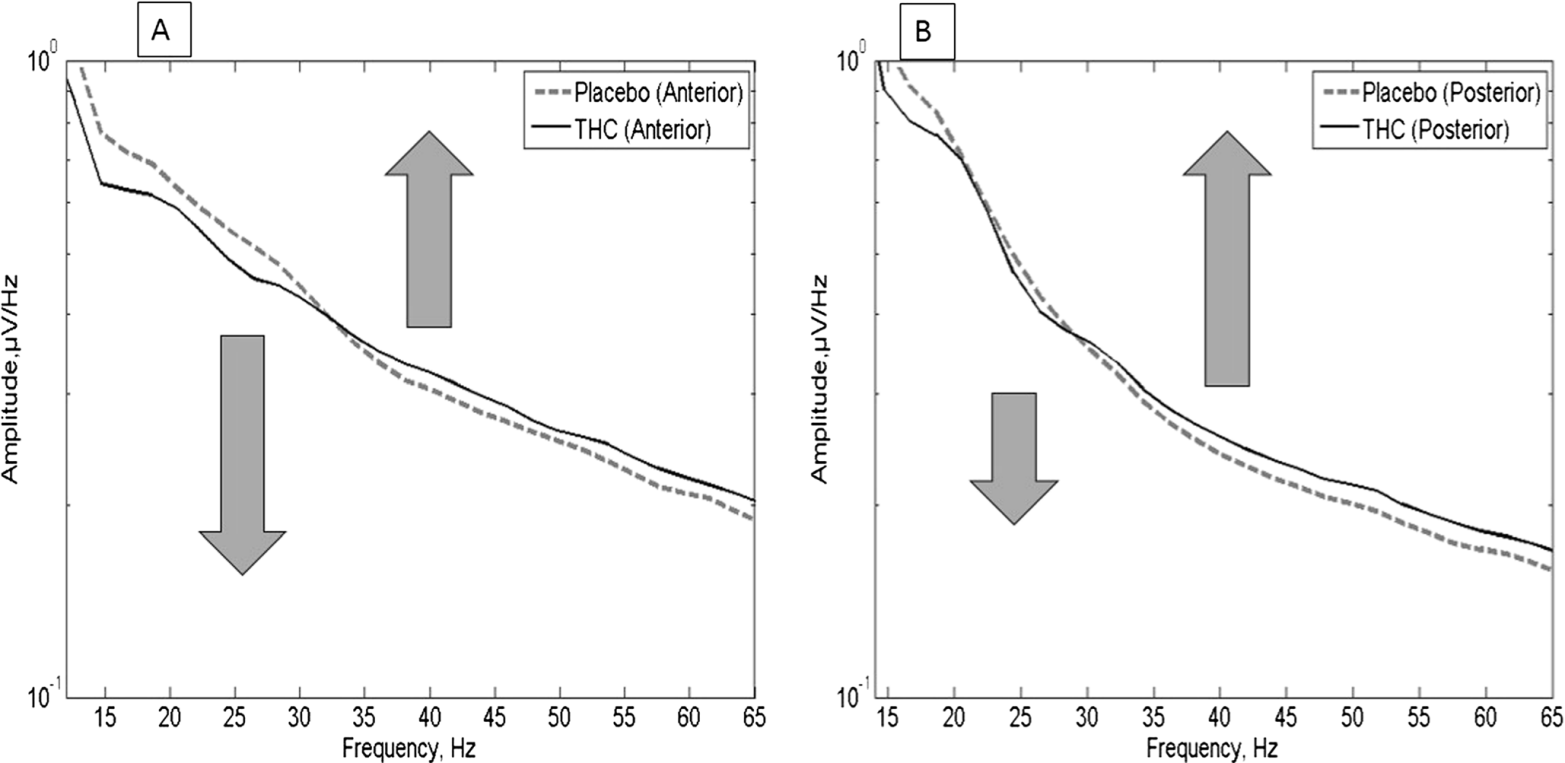
Mean spectra over all subjects: **a** anterior and **b** posterior. The THC-induced shift in magnitude from below 27 Hz to above 27 Hz is visible in both pairs of spectra

**Fig. 3:**
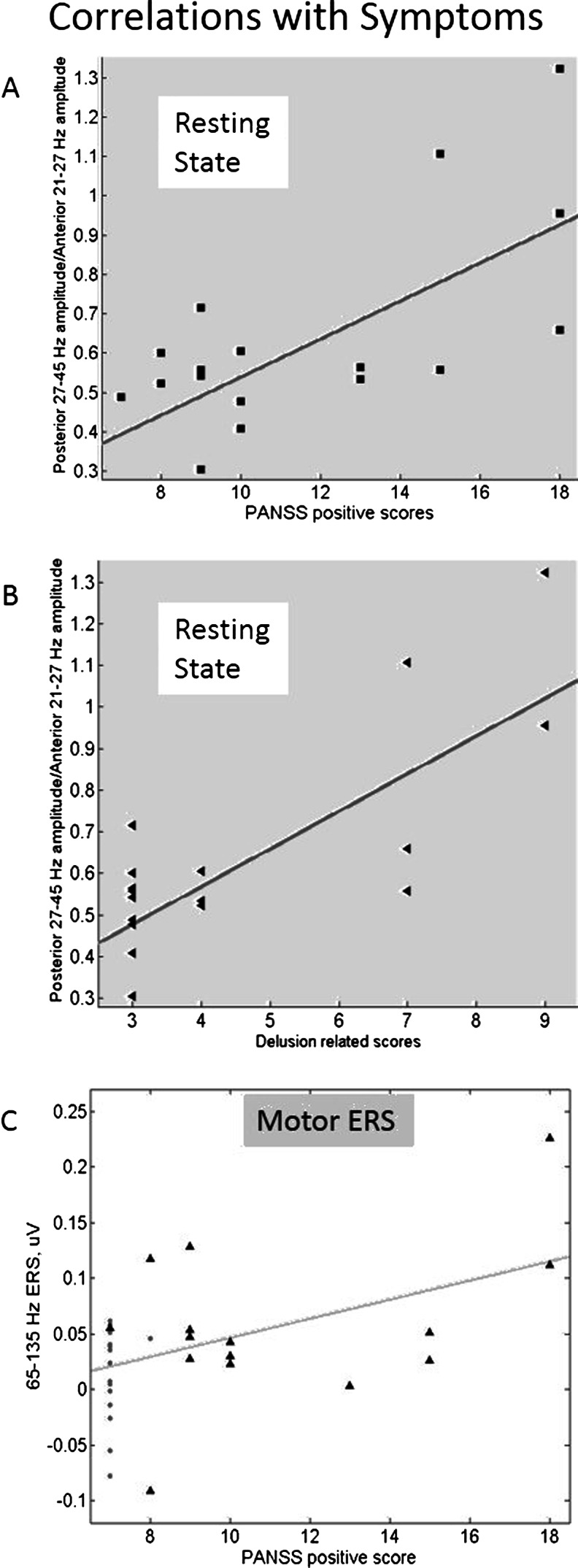
PANSS scores and observed effects. **a**, **b** Association with the ratio of posterior 27–45-Hz magnitude to frontal 20–27-Hz magnitude in the resting state. **a** Total positive scores. **b** Total delusion-related scores: P1, P5 and P6. **c** Motor gamma ERS and positive symptoms. *Gray dots* are placebo sessions and *black triangles* are THC sessions. The *trend line* was fitted for THC sessions only

We did not observe a main effect of condition in the 21–45 Hz band, (*F* = 0.226, *p* = 0.634, *η* ^2^_partial_ = 0.018), but we did find a significant main effect of frequency (*p* < 0.0001) and a condition × frequency interaction (*F* = 15.90, *p* = 0.00017, *η* ^2^_partial_ = 0.570). This effect consisted of an upwards shift in frequency from the 21–27-Hz band to the 27–35 and 35–45-Hz bands under THC, which can be seen in the mean spectra in Fig. 2. There was also a condition × frequency × location interaction (*F* = 6.32, *p* = 0.006, *η* ^2^_partial_ = 0.345). The THC-induced reduction in 21–27-Hz amplitude was relatively greater in frontal areas, whereas the increase in 27–45-Hz magnitude tended to be relatively greater in posterior regions. To explore the relationship between these frequency and spatial effects and symptom scores, two ratios were calculated. Firstly, the overall mean 27–45-Hz amplitude was divided by the 21–27-Hz amplitude, but this first ratio was not correlated with PANSS positive symptom scores. Secondly, to take into account the location effect as well as the frequency effect, the posterior 27–45-Hz amplitude was divided by the anterior 21–27-Hz amplitude (Table 1). This ratio did show a positive association with PANSS positive symptom scores (*r* = 0.429, *p* = 0.021) (see Fig. 3a) in the THC condition, which was still significant after correction for multiple comparisons (*p* = 0.042). Within this correlation with positive symptom scores, the strongest association was with delusion-associated scores (PANSS items P1 + P5 + P6) (*r* = 0.525, *p* = 0.016, after correction for multiple comparisons) (see Fig. 3b).

**Table 1:** Resting-state amplitudes

Mean (SD) μV/Hz (Hz)	Anterior		Posterior	
	Placebo	THC	Placebo	THC
21–27	0.548 (0.190)	0.495 (0.208)	0.506 (0.133)	0.484 (0.149)
27–35	0.419 (0.159)	0.408 (0.178)	0.337 (0.066)	0.345 (0.114)
35–45	0.306 (0.079)	0.323 (0.089)	0.244 (0.057)	0.259 (0.862)

### Self-paced motor task: high-gamma ERS

**Fig. 4:**
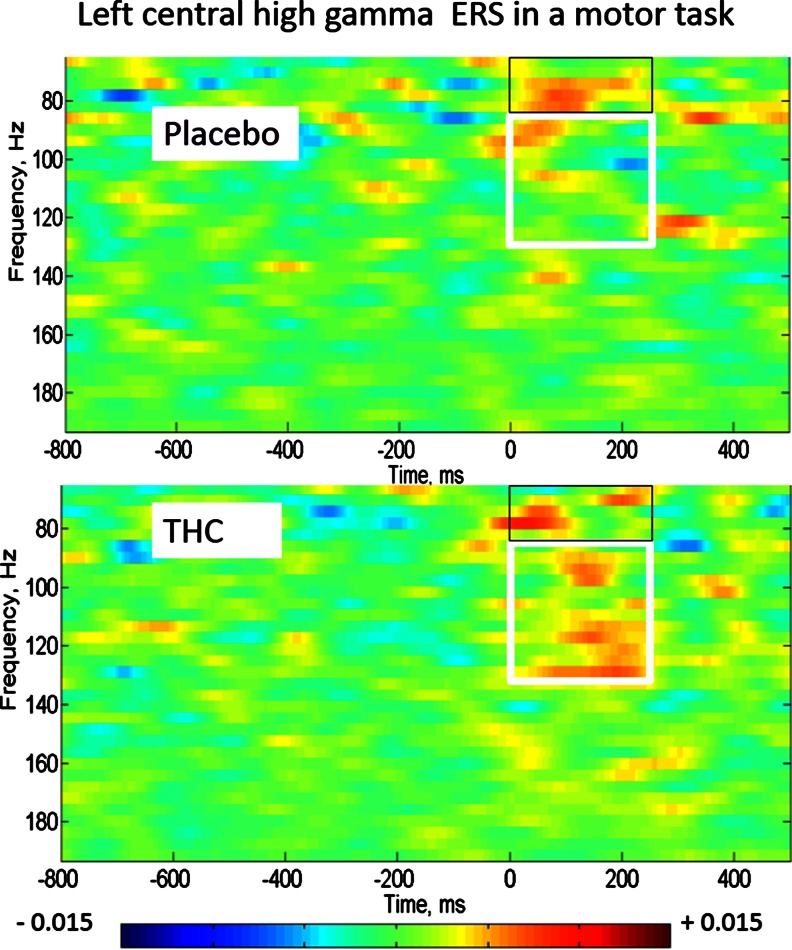
Motor event-related synchronisation at a left central location (C3-CZ). The *white box* highlights the THC-induced increase in 85–130-Hz ERS in the 250 ms after the button press, whilst the *black box* outlines the 65–85-Hz ERS. The amplitudes have been baseline corrected to the 500 ms before the button press. Scale is in μV/Hz

The 65–130-Hz ERS was significantly greater with THC than placebo (*p* = 0.035). To explore the frequency distribution of this effect, using *t* tests with the two-frequency bands separately, a significant increase was observed in the 85–130-Hz band only (placebo −0.002 μV (SD 0.030), THC 0.031 μV (SD 0.033), *p* = 0.02, after correcting for multiple comparisons) but not in the lower-frequency band 65–85 Hz μV (placebo 0.014 (SD 0.020), THC 0.026 μV (SD 0.046), *p* = 0.339, before correction for multiple comparisons) (see Fig. 4).

The distribution of ERS values against PANSS positive scores is shown in Fig. 3c. The correlation between PANSS positive scores and the magnitude of the gamma ERS of the broad (65–130 Hz) frequency band was not significant. However, the increase in gamma was driven by the seven subjects who had detectable delusional symptoms, since repeating the *t* tests with only these subjects still showed a significant increase in ERS with THC in both the 65–130-Hz band (*p* = 0.023) and in the higher, 85–130-Hz sub-band (*p* = 0.036 after correction for multiple comparisons).

## Discussion

### The effect of intravenous THC on the EEG

This study set out to investigate the effects of intravenous THC on high-frequency EEG activity during the resting-state and self-paced motor responses. The novel aspect of the analysis presented here is the application of artefact reduction methods to allow the higher frequencies of the scalp EEG to be investigated with greater confidence. Intravenous THC was found to shift the resting-state spectra towards an increase in the higher-frequency component in the high-beta to low-gamma band and to increase the higher-frequency sub-band of the motor-associated high gamma. In the resting state, an association with PANSS positive symptom scores, particularly scores for delusions, grandiosity and persecution/suspiciousness, was observed.

### Resting-state EEG magnitude effects induced by THC

The previously reported reduction in resting theta with THC (Bocker et al. 2010) was present in our data (Supplement 1). However, our observed magnitude reductions in frequencies between 20 and 27 Hz with THC are difficult to compare directly with previous reports, which do not focus on the same frequency bands, and Böcker and colleagues observed complex, dose-dependent changes in the power of resting ‘beta’ with 29 to 69 mg of smoked THC at mastoid referenced midline electrodes. (Bocker et al. 2010). However, this was in a frequency range of 12–30 Hz, and the spectra shown in their Fig. 6 indicate that power is increased above around 24 Hz but that this is partly outweighed by a decrease in power between 12 and 20 Hz, consistent with our high-frequency resting results. Zuurman and colleagues also reported a reduction in 11.5–30-Hz beta using inhalational THC (Zuurman et al. 2008) in a posterior bipolar channel.

### Comparison with animal studies

Our finding of increased gamma magnitude in the THC sessions appears to differ from reports of the effect of CB1 agonists in rodents, where in the hippocampus, prefrontal and entorhinal cortex, the gamma magnitude is generally decreased (Robbe et al. 2006; Hajos et al. 2008; Kucewicz et al. 2011). However, the contribution of hippocampal electrical activity to human scalp EEG recordings is minimal, and therefore, the effects on neocortical gamma will be more relevant. Also, it should be noted that our experiments were carried out less than an hour after the THC injection and that by 2 h, most of the psychotic symptoms had faded. However, in temporal areas in freely moving rats, decreased gamma magnitude was not apparent until 2 h after the injection (Hajos et al. 2008; Kucewicz et al. 2011). To the contrary, in the entorhinal cortex during the first hour post-injection, the power of both low (30–50 Hz) and high gamma (62–90 Hz) increased (Hajos et al. 2008), which is consistent with our results in humans. However, timing cannot explain the differences in prefrontal areas, since, in a recent animal study, a CB1 agonist induced a reduction in prefrontal gamma magnitude in rats during the first hour after injection (Kucewicz et al. 2011). Whilst the prefrontal regions and spatial scale of the local field potentials sampled by the rat electrodes will not be identical to human prefrontal scalp EEG and different CB1 agonists were used, our results might be due to a genuine species difference in the effect of CB1 agonists on prefrontal gamma (Kucewicz et al. 2011). However, it should also be noted that the increase in gamma in our data was less pronounced in frontal than other regions.

### High-frequency neural oscillations and acute psychosis

It has been previously reported that frequencies above 22 Hz in posterior electrode locations are increased during THC-induced hallucinations compared to the non-hallucinating state, although the effect of scalp and neck muscle activity on these results is unclear (Koukkou and Lehmann 1976). Both ketamine, which can induce psychosis at sub-anaesthetic doses in humans, and MK-801 give rise to a hypersynchronous and persistent 30–80-Hz gamma noise in the parietal, occipital and frontal cortices in awake rats (Hakami et al. 2009), and ketamine also affects oscillations in the rodent basal-ganglian motor system (Nicolas et al. 2011). In an animal model of schizophrenia, but not in controls, ketamine has been shown to produce a peak in gamma above 130 Hz in motor systems (Phillips et al. 2012), which may be related to our finding of an increase in the higher frequency component of motor gamma. Furthermore, amphetamine induces a shift from resting 50 to 80–100 Hz in rat ventral striatum (Berke 2009). This suggests the possibility that the shift to higher frequencies might be a general correlate of agents which induce psychosis. It is of interest that, in addition to much research activity around schizophrenia and gamma, increases in cortical gamma have also been implicated in the induction of false sensory perceptions in humans, such as in tinnitus, somatic hallucinations (Baldeweg et al. 1998) and visual illusions (Adjamian et al. 2004; Weisz et al. 2007). In fact in intracranial stimulation of the tinnitus, hotspot suggests that there may be a causal relationship between tinnitus and EEG changes in the gamma frequency (De Ridder et al. 2011).

### High frequencies in motor systems

CB1 receptors are dense in many components of motor systems, including the cerebellum (Herkenham et al. 1990). The relationship between high-frequency oscillations and pathology in motor systems has been much explored with regard to Parkinson’s disease. Akinesia, in Parkinson’s, has been argued to be due to an abnormally persistent beta 15–30-Hz oscillation in motor systems, whilst oscillations at frequencies above 70 Hz are thought to be pro-kinetic (Brown and Williams 2005; Schnitzler and Gross 2005) This high-frequency gamma is disinhibited by the dopamine agonists used to treat the disease, which can also induce psychosis. Our observation that THC also increases high-frequency motor gamma magnitude suggests that these two drug effects might be related. Also, it is of interest that deep brain stimulation of the sub-thalamic nucleus, typically at 130 Hz, is increasingly used to treat Parkinson’s patients. There is evidence that the mechanism of this effect is antidromic conduction of these 130-Hz oscillations back to layer V pyramid cells in the motor cortex (Dejean et al. 2009; Gradinaru et al. 2009). Our observed shift from beta to gamma in the resting state might be interpreted as an activation of cognitive systems by THC which is analogous to the high-frequency activation of motor systems by dopamine agonists.

### Possible mechanisms for the frequency shift

There are two possible levels of explanation for the upwards frequency shift: either it is a direct effect within the neocortex or it is a secondary effect mediated via sub-cortical structures and feeding back on to the cortex. On the neocortical level, CB1 receptors inhibit GABA release from CCK-immunoreactive, irregular spiking inter-neurons (Bodor et al. 2005; Galarreta et al. 2008), which could be expected to produce an increase in excitation of the pyramid cell and parvalbumin-immunoreactive fast spiking inter-neuron ‘PING’ network responsible for generating neocortical gamma (Whittington et al. 2011), as previously pointed out by others (Robbe et al. 2006). Increasing the excitatory inputs to such a network can increase the frequency of the oscillations (see Fig. 1b in Whittington et al. (2011)). If the peak frequency of gamma is an index of saliency (van Pelt and Fries 2013), then this would imply that THC induces both aberrant salience and delusions (Kapur 2003) by a direct action on the cortex. However, this would be inconsistent with research implicating sub-cortical D2 signalling in delusion formation in schizophrenia (Howes and Kapur 2009).

How might the THC-induced increase in higher frequency neural oscillations be mediated via a sub-cortical pathway? As discussed above, in the motor basal ganglia, THC produces similar effects to dopamine on neural oscillations. It could therefore be speculated that the process involves dopamine-like effects in non-motor regions of the basal ganglia, such as the nucleus accumbens (NA) or caudate nucleus. Information processing in these basal ganglia areas might be impaired by THC-induced theta desynchronisation of hippocampal and the prefrontal afferents on to these regions (Goto and Grace 2008; Morrison et al. 2011). This could produce a feed forward deficit in the information transmitted to areas which induce or synchronise cortical gamma and beta oscillations, such as cholinergic centres (Metherate et al. 1992; Verrico et al. 2003; Roopun et al. 2010), particularly the pedunculopontine nucleus, or intralaminar or posterior thalamic nuclei. Functional basal ganglia-tecto-thalamic circuits are known to exist, including connections with gamma-inducing areas (Leh et al. 2008; Redgrave et al. 2010). Motor gamma has been postulated to be dependent on the centromedian and parafascicular nuclei of the thalamus (Jenkinson et al. 2013) whereas the pulvinar may mediate the resting-state effects (Bouyer et al. 1981; Shumikhina and Molotchnikoff 1999). The pulvinar, which in cats synchronises visual gamma and beta oscillations (Shumikhina and Molotchnikoff 1999), cannot be identified in rodents and has connections with the prefrontal and temporal areas in primates (Romanski et al. 1997), including humans (Leh et al. 2008). Involvement of the pulvinar would explain the difference in the effect of CB1 agonists on prefrontal gamma power in rodents (Kucewicz et al. 2011) and humans. Also, the pulvinar is thought to mediate salience (Robinson and Petersen 1992), and delusions can be induced by a lesion in the pulvinar (Crail-Melendez et al. 2013).

Whatever the cause of the upward shift in frequency, it is of interest that we found resting-state beta, which has a function to maintain the status quo (Engel and Fries 2010), being replaced by gamma, which is associated with the active processing of signals (Fries et al. 2001; Sohal et al. 2009). This gamma over-activity may lead to neuronal noise being given excessive processing, leading to false inferences. In higher-order brain regions, this may produce erroneous interpretations of the social environment which are manifested in a THC-induced paranoid delusion.

In summary, at frequencies above 20 Hz, intravenous THC increases the magnitude of higher-frequency components, especially in motor and posterior cortex. These changes correlate with positive symptom scores on the PANSS scale. The shift from lower to higher frequencies under THC may reflect a cortical over-activation which could underlie the induction of positive symptoms by THC.

## Electronic supplementary material

Below is the link to the electronic supplementary material.

ESM 1 (DOCX 14 kb)
