# Supplementary material for: Delta-9-tetrahydrocannabinol, neural oscillations above 20 Hz and induced acute psychosis
Source: Psychopharmacology (Berl). 2014 Jul 20;232(3):519–28. doi: 10.1007/s00213-014-3684-1 (PMC4302232; doi:10.1007/s00213-014-3684-1)
Supplement: Supplementary file 1 — (DOCX 14 kb) [file 213_2014_3684_MOESM1_ESM.docx]

# **Delta-9-Tetrahydrocannabinol, neural oscillations above 20 Hz and induced acute psychosis.**

**Psychopharmacology**

Judith F. Nottage^1^, James Stone^1^, Robin M Murray^1^, Alex Sumich^2^, Elvira Bramon-Bosch^3^, Dominic ffytche^1^* and Paul D Morrison^1^*

^1.^ King’s College London, Institute of Psychiatry, ^2^Nottingham Trent University, ^3.^University College London

**Supplement 1:**

**The effect of intra-venous THC on low frequency resting EEG**

**Methods:** Low frequencies (0.5-20 Hz) were analysed using standard Neuroscan software. Blink artifacts were corrected using regression with the VEOG channel. Epochs of length 2048 ms, were used with 10% overlap, and artefactual segments were manually rejected. Fast Fourier transforms (FFT) were carried out to obtain amplitudes values, using the Neuroscan 10% Hanning window.The frequencies below 20 Hz were divided into the following bands: low delta (0.5-1.0 Hz), delta (1.0-3.4 Hz), theta (3.4-8.3 Hz) low alpha (8.3-9.8 Hz) high alpha (9.8-13.2 Hz) and low beta (13.2-19.5 Hz). Eight regions were investigated: left and right prefrontal, left and right central, left and right temporal, left and right occipito-parietal. Repeated measures ANOVAs were carried out for the amplitudes in each frequency band. The statistical models used included condition, region and laterality.

**Results:** Delta, theta and high alpha amplitudes were all significantly reduced (see Table ), although the delta effect does not survive correction for multiple comparisons. There was also a condition x region interaction in theta (p=0.009), due to the THC induced theta reduction being greatest in frontal regions.

Table S1: Low frequency amplitudes

| **Frequency band** | **p value for drug effect** | **Mean Placebo**  **(μV)** | **Mean THC**  **(μV)** |
| --- | --- | --- | --- |
| Low delta | 0.245 | 2.518 | 2.327 |
| Delta | 0.019 | 1.827 | 1.630 |
| Theta | 0.002 | 1.187 | 0.980 |
| Low alpha | 0.166 | 1.581 | 1.368 |
| High alpha | 0.009 | 1.252 | 1.082 |
| Low beta | 0.431 | 0.740 | 0.711 |

**Note:** p values are not corrected for multiple comparisons
